# Qualitative and quantitative three-dimensional evaluation of maxillary basal and dentoalveolar dimensions in patients with and without maxillary impacted canines

**DOI:** 10.1186/s40510-022-00434-3

**Published:** 2022-10-24

**Authors:** Hasan M. Sharhan, Abeer A. Almashraqi, Hanan Al-fakeh, Najah Alhashimi, Ehab A. Abdulghani, Wenyuanfeng Chen, Abeer A. Al-Sosowa, BaoCheng Cao, Maged S. Alhammadi

**Affiliations:** 1 Department of Orthodontics and Dentofacial Orthopedics, College of Dentistry, Lanzhou University, Lanzhou, China; 2 Department of Orthodontics, Faculty of Dentistry, Thamar University, Dhamar, Republic of Yemen; 3 Department of Pre-Clinical Oral Health Sciences, College of Dental Medicine, QU Health, Qatar University, Doha, Qatar; 4 Prosthodontics Department, School of Stomatology, Lanzhou University, Lanzhou, China; 5 Prosthodontics Department, Faculty of Dentistry, Thamar University, Dhamar, Republic of Yemen; 6 Unit and Divisional Chief Orthodontics at Hamad Medical Corporation; College of Dental Medicine, Qatar University, Doha, Qatar; 7 Department of Periodontics, Faculty of Dentistry, Thamar University, Dhamar, Republic of Yemen; 8 Department of Periodontics, College of Dentistry, Lanzhou University, Lanzhou, China; 9 Orthodontics and Dentofacial Orthopedics, Department of Preventive Dental Sciences, College of Dentistry, Jazan University, Jazan, Saudi Arabia; 10 Postgraduate Orthodontic Program, Department of Orthodontics, Pedodontics and Preventive Dentistry, Faculty of Dentistry, Sana’a University, Sana’a, Republic of Yemen

**Keywords:** Impacted canine, Bone quantity, Bone quality, CBCT

## Abstract

**Background:**

This study aimed to three-dimensionally evaluate the qualitative and quantitative maxillary basal, dentoalveolar, and dental dimensions in patients with unilateral or bilateral maxillary impacted canines relative to their normal peers.

**Materials and methods:**

This is a retrospective comparative study. Cone-beam computed tomography images of one hundred and fifty adult patients were divided into three equal groups: unilateral, bilateral, and control groups. Each had 50 patients that were three-dimensionally analysed. The quantitative measurements involved three basal (molar basal width, premolar basal width, and arch depth), seven dentoalveolar (molar alveolar width, premolar alveolar width, inter-molar width, inter-premolar width, inter-canine width, arch length, and arch perimeter), and two dental (canine length and width) measurements. The qualitative measurements included four bone density areas (buccal, lingual, mesial, and distal) around the maxillary impacted canines.

**Result:**

Differences between the three groups were statistically different for the quantitative measurements involving the two basal variables (molar basal width and premolar basal width) and all measured dentoalveolar variables; these were smaller in the unilateral and bilateral groups compared with the control group (*p* < 0.001). Unilateral and bilateral impacted canine groups showed significantly wider and shorter canines than the control group (*p* < 0.001). The qualitative measurements (the four bone density areas) around unilateral and bilateral impacted canine groups showed significantly greater density than the control group (*p* < 0.001). There was no significant qualitative or quantitative difference between the unilateral and bilateral impacted canines. The three groups had no significant variations in terms of arch depth.

**Conclusion:**

Maxillary unilateral and bilateral canine impactions are associated with reduced basal and dentoalveolar dimensions as well as wider and shorter maxillary canines compared to normal peers. The quality of bone around unilateral and bilateral impacted maxillary canines is higher than in non-impacted cases. Unilateral and bilateral canine impactions have quite similar qualitative and quantitative parameters.

## Background

Impacted maxillary canines are a prevalent orthodontic clinical finding; the morphologic differences in the maxillofacial and dentoalveolar tissues could be linked to this dental eruption anomaly [1]. The maxillary canine is the third-most impacted tooth following the maxillary and mandibular third molars [2]. In Caucasian patients, the majority of affected canines are palatally displaced [3], but buccally displaced cases are more common in Asian patients [4]. It has been estimated that 1 to 3% of the general population suffers from maxillary persistent canine impaction [5], with unilateral cases being more prevalent than bilateral impaction [6]. Female patients are more likely to experience impaction, and some researchers report that it occurs twice as frequently as male patients [5, 6].

At least two theories characterize the aetiology of palatally impacted canines: genetic theory [7] and guiding theory [8]. The genetic theory implicates genetic factors as the primary cause of palatally displaced maxillary canines as well as other potentially related dental abnormalities [3]. According to guiding theory, the canine erupts along the root of the lateral incisor, which acts as a guide. The canine will not erupt if the root of the lateral incisor is missing or deformed [9]. In contrast, dental crowding appears to be the cause of labially impacted canines [10]. Palatally displaced canines are two to three times more common than labially impacted canines [5].

It was reported that the sequelae of canine impaction included: malposition of the impacted tooth, either labial or lingual, adjacent tooth migration, arch length loss, internal resorption, formation of dentigerous cyst, external root resorption of the impacted tooth and adjacent teeth, infection, especially with partial eruption, as well as referred pain and combinations of the aforementioned sequelae [11]. These potential issues highlight the necessity of careful monitoring of the development and eruption of these teeth during “normal” periodic dental examinations of the developing child [12].

The treatment of impacted canines is crucial for both aesthetics and function. Clinicians must develop treatment plans that are in the best interests of the patient and be knowledgeable about the various therapy alternatives. When patients are correctly examined and treated, clinicians can reduce the occurrence of ectopic eruption and subsequent maxillary canine impaction [12].

The relationship between impacted maxillary canines and maxillary morphology including the maxillary transverse dimension is controversial and, at times, contradictory [13]. McConnell et al. linked a transverse maxillary deficiency to palatally displaced canines [14, 15], which contrasts with other research showing a link between larger maxillary transverse dimensions and canine impaction [16]. However, these studies did not match the groups to establish accurate comparisons. Other authors found no significant variations in maxillary width [17–19], although their research lacked a sufficient control group. Thus, it becomes crucial to determine if impacted maxillary canines are related to maxillary transverse skeletal dimensions as well as the dentoalveolar dimensions and the surrounding bone quality.

The craniofacial growth and development during the adolescent age might result in changes in the jaw dimensions; for this reason it is recommended to conduct studies of this nature on adults’ patients [20].

To date, no study has yet compressively evaluated the qualitative and quantitative measurements of the impacted maxillary canines. The null hypothesis was that there are no differences between the quantitative maxillary basal, dentoalveolar, and dental dimensions and the qualitative bone surrounding in patients with unilateral or bilateral impacted maxillary canines relative to their normal peers. Thus, this study aimed to three-dimensionally evaluate the qualitative and quantitative maxillary basal, dentoalveolar, and dental dimensions in patients with unilateral or bilateral maxillary impacted canines compared to their normal peers.

## Materials and methods

### Study design and participants

This is a retrospective cross-sectional study that was approved by the ethics committee, Hospital of Stomatology, Lanzhou University, China (No LZUKQ-2022-025). The sample was collected from out-patient clinic records at the Hospital of Stomatology, Lanzhou University, China. The subjects were divided into three equal groups: unilateral maxillary canine impaction, bilateral maxillary canine impaction, and control groups (no impaction). The inclusion criteria included the following: (1) age between 16 and 30 years old; (2) presence of unilateral or bilateral maxillary impacted canines; (3) full set of erupted teeth with/without the third molars; (4) absence of the deciduous maxillary canines; and (5) scans with good image definition. The exclusion criteria were: (1) crown/bridge or interproximal caries or restorations; (2) presence of aggressive or progressive periodontitis; (3) previous orthodontic treatment; (4) dental agenesis, maxillary lesions, trauma, or tumours; (5) cleft palate or lip, craniofacial abnormalities, (6) presence of hyper- or hypodontia; (7) neck and head disorders; and 8) patient with reported systemic bone disease.

### Study sample size

The sample size was determined utilizing G*power 3.0.10 software with an alpha value of 0.05 and a power of 90% based on the study conducted by Arboleda-Ariza et al. [18], who reported first molars’ alveolar width of 54.2 ± 4.2 mm and 57.2 ± 2.7 mm in the unilateral impacted canines and control group, respectively. Another calculation was considered using a variable representing the vertical growth pattern (NSAr angle) in which the reported values were 126.5 ± 5.9 and 122.6 ± 5.7 mm in unilateral impaction and control groups, respectively. Power analysis based on the two calculations showed a minimum sample of 31 and 48 subjects, respectively. However, the sample size was increased to 50 patients for each study group.

### Three-dimensional imaging

Three-dimensional images were acquired using a CBCT (I-CAT®; Imaging Sciences International, Hatfield, PA). CBCT imaging parameters were set at 120 kV, 16.0 cm × 13 cm field of view, and 8.9 s of exposure time with a voxel size of 0.3 mm and a slice thickness of 0.5 mm. The patients were sitting upright with their teeth close to their maximum intercuspation. The Frankfort horizontal plane was positioned parallel to the floor, and the midsagittal plane was perpendicular to the floor; all patients were instructed not to swallow during scanning. The collected CBCT scan data were transferred into a DICOM (Digital Imaging and Communication in Medicine) file format and then imported into the In vivo 6 software (Anatomage, San Jose, CA, USA) for 3D analysis.

### Outcomes assessment

A series of cross-sectional views were obtained in secondary reconstruction mode, and the bone of the jaw was aligned parallel to the reference surface using the orientation coordinates. Molar measurements were taken on the most anterior coronal slice of the CBCT scan with the palatal plane horizontal, thus demonstrating the buccal root furcation. Landmarks on the right and left nasal floors were positioned in the most inferior location to establish a nasal floor reference line running between these two landmarks. The same landmark placement and reference line were used for premolar measurements on the coronal slice, thus revealing the centre of the root canal for molar measurements.

**Fig. 1:**
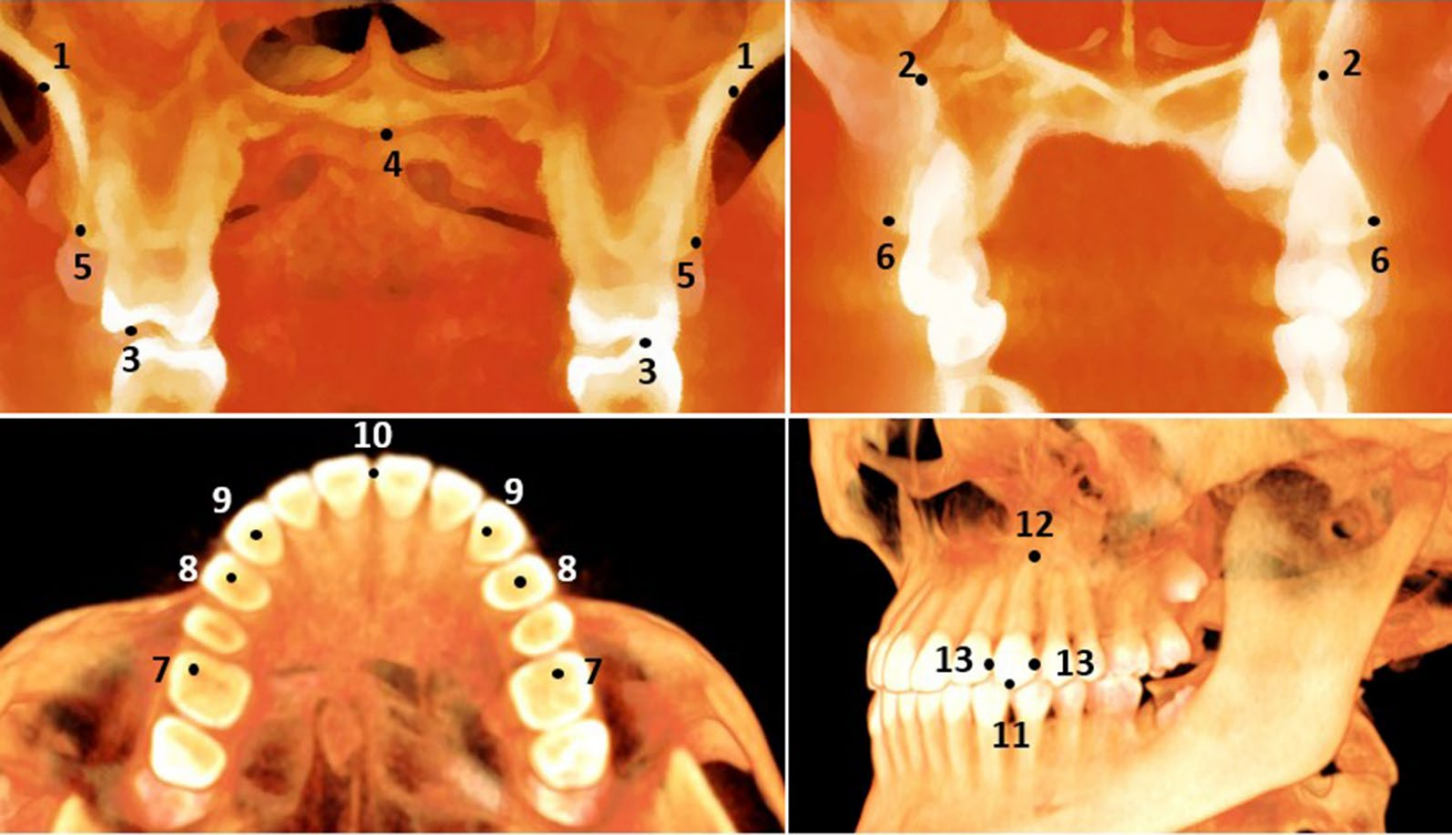
Three-dimensional views of the selected landmarks used in the study

**Fig. 2:**
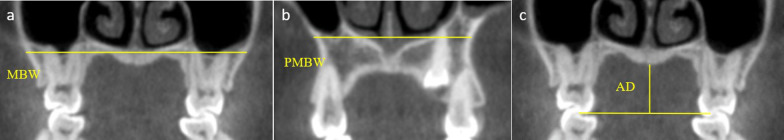
The quantitative basal measurements: **a** MBW, first molar basal width; **b** PMBW, first premolar basal width; and** c** AD, arch depth

The landmarks used are presented in Table 1 and Fig. 1. The outcomes were divided into quantitative and qualitative measures. The quantitative measurements included three basal, seven dentoalveolar, and two dental measurements (Table 2). The basal measurements included molar basal width (MBW), first premolar basal width (PMBW), and arch depth (AD) (Fig. 2).

**Table 1:** Definitions of the anatomical three-dimensional: skeletal, dentoalveolar, and dental landmarks used in the study

No	Landmark	Definition
*Quantitative basal landmarks*		
1	Molar Right/Left Nasal Floor	The most inferior point on the right/left side of the nasal floor at the level of maxillary first molars
2	Premolar Right/Left Nasal Floor	The most inferior point on the right/left side of the nasal floor at the level of maxillary first premolars
3	Right/Left Molars Buccal Cusp	The most inferior point of the right /left buccal cusps at the centre of the maxillary first molars
4	Mid-palatal Point	The most inferior point of the oral floor of the palatal bone at the level of the maxillary first molars
*Quantitative dentoalveolar landmarks*		
5	Alveolar molar point	The most infero-lateral point on the alveolar ridge opposite the centre of the maxillary first molar
6	Alveolar premolar point	The most infero-lateral point on the alveolar ridge opposite the centre of the maxillary first premolar
7	Molar Dental Point	The point on the right/left side of the mesiobuccal cusp at the centre of the maxillary first molars
8	Premolar Dental Point	The point on the right/left side of the buccal cusp at the centre of the maxillary first premolars
9	Canine Dental Point	The point on the right/left side at the centre of the maxillary canines
10	Midline point	The mesial contact point on the right/left side of the maxillary incisors
*Quantitative dental landmarks*		
11	Canine Cusp Tip	The most inferior point on the centre of the cusp of the maxillary canines
12	Canine Root Apex	The most superior point on the centre of the root of the maxillary canines
13	Contact Point	The most lateral point on the mesial/distal contact of the maxillary canines

**Table 2:** Definitions of the measurements used in the study

Variable	Definition
*Quantitative basal measurements*	
MBW	The maxillary first molar basal width dimension was measured along the nasal floor reference plane, following the line created by the outside corners of the right and left sides of the maxillary base (lateral limits)
PMBW	The maxillary first premolar basal width dimension was measured at the nasal floor reference plane along a line formed by the outside edges of the right and left sides of the maxillary base (lateral limits)
AD	Arch depth was equally distant between the inter-molar line (right /left buccal cusps at the centre of the maxillary first molars) and the mid-palatal point
*Quantitative Dentoalveolar measurements*	
MAW	The maxillary first molar alveolar width measure was measured on the first molar coronal slice between the most occlusal sites of the maxillary alveolar process
PMAW	The maxillary first premolar alveolar width was measured on the first premolar coronal slice between the most occlusal sites of the maxillary alveolar process
IMW	The inter-molar width was determined by measuring the width between the right and left mesiobuccal cusp at the centre of the maxillary first molars
IPW	The inter-premolar width was determined by measuring the width between a set position in the right and left buccal cusps at the centre of the maxillary first premolars
ICW	The inter-canine width was determined by measuring the width between the right and left centre of the maxillary canines
AL	The arch length defined as the distance between the mesial contact point of the incisors and the inter-molar plane perpendicular to the midline
AP	The arch perimeter was measured from one side's mesiobuccal cusp of the first permanent molar to the other side's mesiobuccal cusp of the molar
*Quantitative Dental measurements*	
CL	The canine length was determined by measuring from the edge of the incisal to the root apex
CW	The canine width was measured from the mesial point of contact to the distal point of contact

**Fig. 3:**
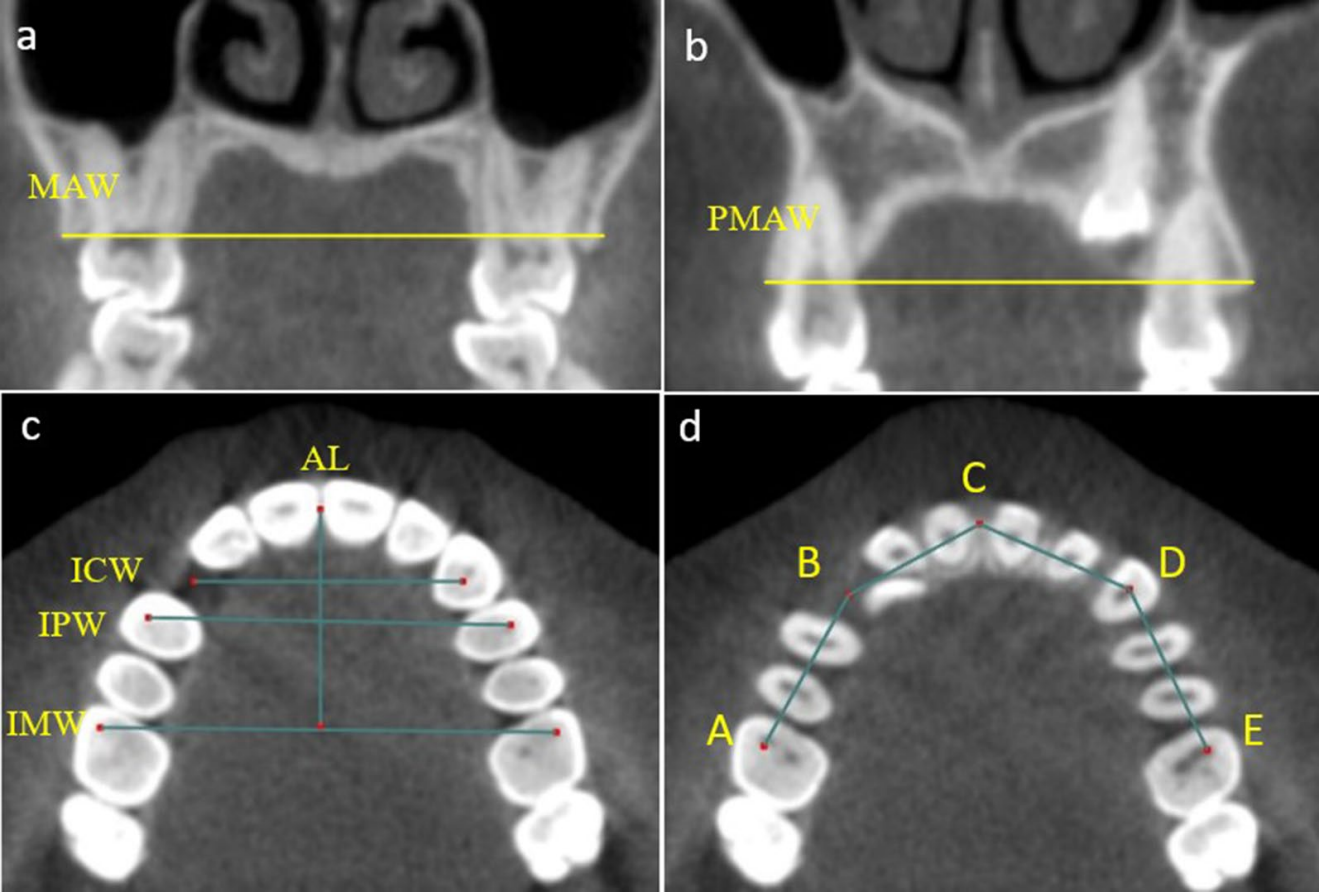
The quantitative dentoalveolar measurements: **a** MAW, first molar alveolar width; **b** PMAW, first premolar alveolar width; **c** IMW, inter-molar width; IPMW, inter-premolar width; ICW, inter-canine width; and AL, arch length; **d** Arch perimeter: the sum of distances A–B, B–C, C–D, and D–E

**Fig. 4:**
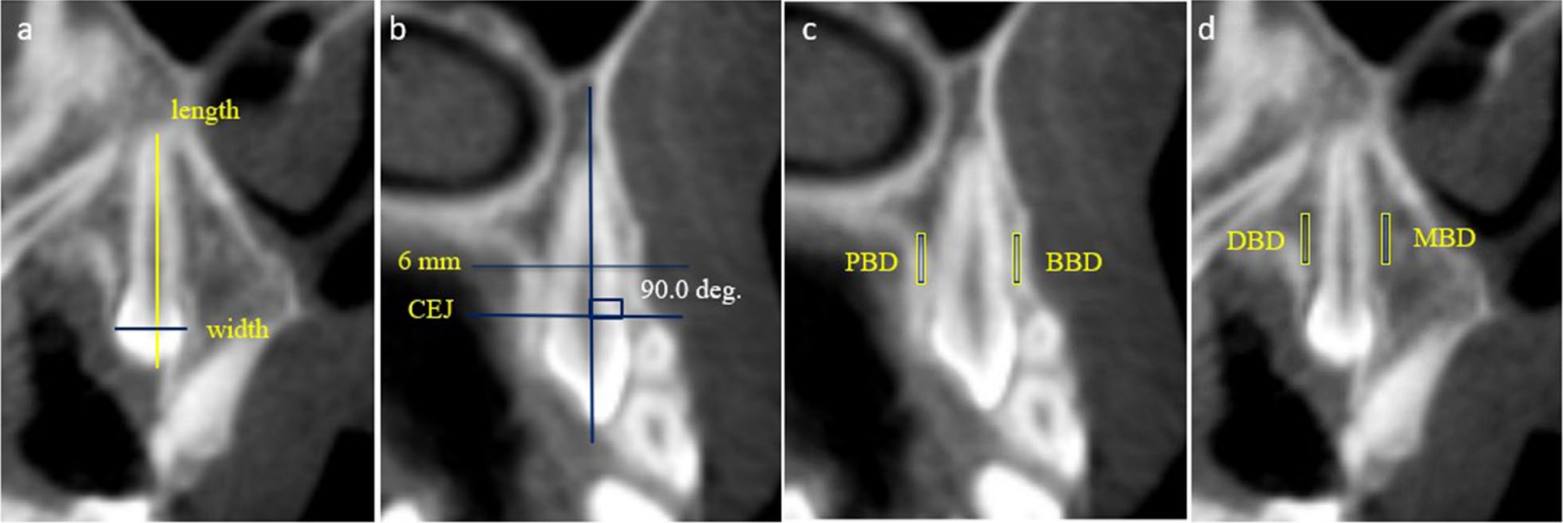
**a** The quantitative dental measurements including length and width of canines; **b** Measurement of bone density around the impacted canine between the CEJ line's midpoint and the root apex. **c**, **d** Measurement of the bone density at each surface mesially, distally, facially, and palatally

The dentoalveolar measurements were the molar arch width (MAW), premolar arch width (PMAW), inter-molar width (IMW), inter-premolar width (IPW), inter-canine width (ICW), arch length (AL), and arch perimeter (AP) (Fig. 3). In addition, the dental measurements involved canine length (CL) and width (CW) (Fig. 4a).

The qualitative measurements included four bone density areas: buccal, palatal, mesial, and distal around the maxillary impacted canines. The average value of bone density around the canine was measured in three regions of interest and at one point in the middle region canine length (Fig. 4b). The value registers in a spot diameter of 1 mm each mesially, distally, facially, and palatally (Fig. 4c, d).

### Statistical analysis

All data were analysed using Statistical Package for Social Sciences (SPSS) 22.0 (software (IBM Corp., Armonk, NY, USA). The means and standard errors, standard deviations, and averages were used to present descriptive statistics. The test of normality used the Shapiro–Wilk’s test and the Kolmogorov–Smirnov test. The one-way ANOVA t test was utilized when comparing the three groups (control, unilateral, and bilateral groups). To determine the significant error of the radiographic measurement technique, random CBCT of 33 patients were re-measured by the primary investigator two weeks after the first measurements and by other well-trained examiners under the direct supervision of a well-experienced oral and maxillofacial radiologist. Reliability and reproducibility were analysed by the Cronbach’s alpha and international classification tests. Finally, the trial was performed with 95% confidence.

## Results

One hundred and fifty subjects with a mean age of 23.09 ± 6 years old were assessed. The mean ages of the male and female groups were 22.9 ± 6.45 and 23.19 ± 5.79 years, respectively. The gender distribution of the selected sample is shown in Table 3.

**Table 3:** Sex and side upper canine impaction distribution in each group

	*N* (%)									
	Control (*n* = 50)		Unilateral impaction (*n* = 50)				Bilateral impaction (*n* = 50)			
	Right (*n* = 50)	Left (*n* = 50)	Right (*n* = 19)		Left (*n* = 31)		Right (*n* = 50)		Left (*n* = 50)	
			Buccal	Palatal	Buccal	Palatal	Buccal	Palatal	Buccal	Palatal
Male	22 (43.1)	22 (43.1)	5 (12.8)	4 (10.3)	5 (12.8)	3 (7.7)	6 (17.6)	6 (17.6)	5 (14.7)	7 (20.6)
Female	28 (58.8)	28 (58.8)	5 (8.2)	5 (8.2)	10 (16.4)	13 (21.3)	26 (40)	12 (18.2)	28 (43.1)	10 (15.2)

The most bilateral group has a buccal impaction. The unilateral group had the same buccal and palatal impaction. Buccal impaction was more common than palatal impaction, which was more common in women than men (Table 3).

The intra-class correlation coefficient (ICC) revealed high consistency (0.977; *p* < 0.001). Table 4 shows that the ICC was excellent; all coefficients of reliability (ICC) values were above the 0.95 proposed cut-off value; this means that the measurement error for this study was low. All values were below the acceptable 5% line, thus indicating a high level of methodology reliability.

**Table 4:** The intra-class correlation coefficient (ICC) for reliability

Measurements	Intra-observer error			Inter-observer error		
	Single measure	Average measures	Cronbach's alpha	Single measure	Average measures	Cronbach's alpha
MBW	0.94	0.97	0.97	0.97	0.98	0.98
PBW	0.97	0.98	0.98	0.87	0.97	0.97
AD	0.80	0.89	0.89	0.83	0.91	0.91
MAW	0.87	0.93	0.93	0.91	0.95	0.95
PMAW	0.81	0.89	0.89	0.88	0.94	0.94
IMW	0.82	0.90	0.90	0.80	0.89	0.89
IPW	0.78	0.87	0.87	0.87	0.93	0.93
ICW	0.84	0.91	0.91	0.84	0.91	0.91
AL	0.80	0.89	0.89	0.89	0.98	0.98
AP	0.91	0.95	0.95	0.88	0.94	0.94
CL	0.93	0.96	0.96	0.82	0.83	0.83
CW	0.82	0.90	0.90	0.83	0.97	0.97
Bone density	0.95	0.97	0.95	0.82	0.89	0.89

**Fig. 5:**
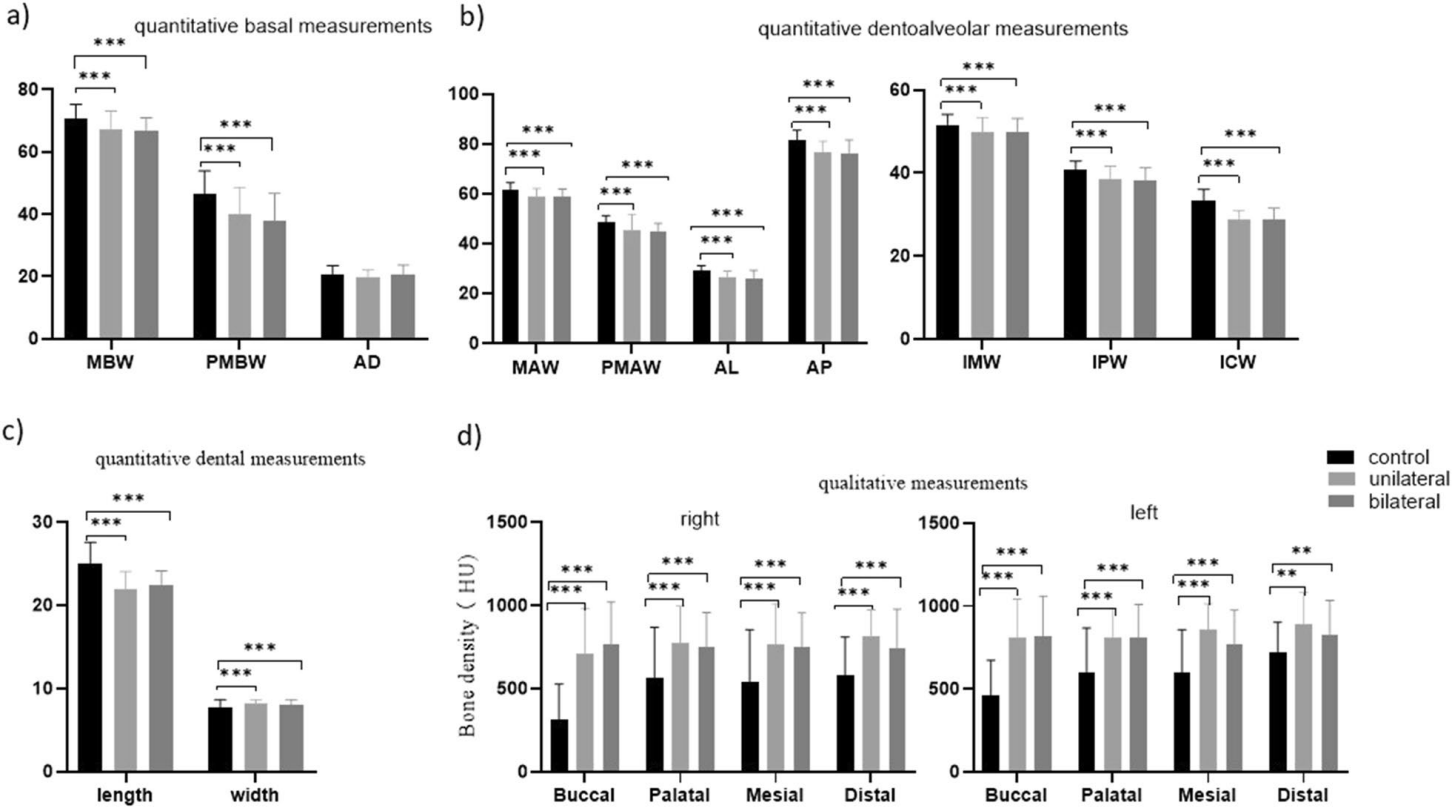
Comparison of the qualitative and quantitative measurements between impacted and control groups

There were significant differences between the three groups for MBW and PMBW for quantitative maxillary basal measurements. These were lesser in the unilateral and bilateral groups compared with the control group (*p* < 0.001). The lowest difference was for the MBW measurement (3.3 mm) between the control (70.70 ± 4.52 mm) and the unilateral (67.37 ± 5.75 mm) groups. The highest difference was for the PMBW measurement (8.7 mm) between the control (46.54 ± 7.39 mm) and bilateral (37.79 ± 8.88 mm) groups. There were no significant differences between the three groups related to arch depth (Table 5; Fig. 5a).

**Table 5:** Comparison of quantitative basal, dentoalveolar, and dental measurements between control and impaction groups

	Mean ± SD	Mean ± SD	Mean ± SD	*p* value
	Control (*n* = 50)	Unilateral (*n* = 50)	Bilateral (*n *= 50)	
Quantitative basal measurements				
MBW	70.70 ± 4.52^a^	67.37 ± 5.75^b^	66.69 ± 4.25^b^	< 0.001
PMBW	46.54 ± 7.39^a^	40.20 ± 8.34^b^	37.79 ± 8.88^b^	< 0.001
AD	20.77 ± 2.74	19.78 ± 2.34	20.54 ± 3.13	0.170
Quantitative dentoalveolar measurements				
MAW	61.71 ± 2.85^a^	58.84 ± 3.29^b^	58.94 ± 2.91^b^	< 0.001
PMAW	48.68 ± 2.60^a^	45.32 ± 6.43^b^	44.89 ± 3.33^b^	< 0.001
IMW	51.64 ± 2.48^a^	49.82 ± 3.55^b^	49.94 ± 3.20^b^	< 0.001
IPW	40.83 ± 2.05^a^	38.55 ± 3.06^b^	38.14 ± 3.09^b^	< 0.001
ICW	33.26 ± 2.76^a^	28.68 ± 2.12^b^	28.85 ± 2.67^b^	< 0.001
AL	29.00 ± 2.22^a^	26.33 ± 2.72^b^	26.23 ± 3.02^b^	< 0.001
AP	81.65 ± 3.91^a^	76.54 ± 4.61^b^	75.95 ± 5.68^b^	< 0.001
Quantitative dental measurements				
CL all	24.98 ± 2.59^a^	21.91 ± 2.15^b^	22.39 ± 1.74^b^	< 0.001
CL Right	24.74 ± 2.63^a^	22.08 ± 2.56^b^	22.35 ± 1.74^b^	< 0.001
CL Left	25.22 ± 2.56^a^	21.81 ± 1.89^b^	22.43 ± 1.76^b^	< 0.001
CW all	7.77 ± 0.90^a^	8.16 ± 0.50^b^	8.12 ± 0.57^b^	< 0.001
CW Right	7.95 ± 0.85^a^	8.14 ± 0.49^b^	8.11 ± 0.56^b^	0.014
CW left	7.79 ± 0.96^a^	8.16 ± 0.54^b^	8.14 ± 0.58^b^	0.048

^a, b^ Letters used for significance differences between studied groups; similar letter indicated insignificant difference while different letters indicated significant differences between studied groups

For quantitative dentoalveolar measurements, the results showed significant differences between the three examined groups for MAW, PMAW, IMW, IPW, ICW, AL, and AP assessment; these were lesser in the unilateral and bilateral groups versus the control group (*p* < 0.001). The lowest difference was for the IMW measurement (1.7 mm) between the control (51.64 ± 2.48 mm) and the bilateral (49.94 ± 3.20 mm) groups. The highest difference was for the ICW measurement (4.5 mm) between the control (33.26 ± 2.76 mm) and unilateral (28.68 ± 2.12 mm) groups (Table 5; Fig. 5b).

For AL and AP assessment, the results showed that there were significant differences between the three examined groups: these were lesser in the unilateral and bilateral groups than in the control group (*p* < 0.001). The lowest difference was for the AL measurement (2.6 mm) between the control (29 ± 2.22 mm) and the unilateral (26.33 ± 2.72 mm) groups. The highest significant difference was for the AP measurement (5.7 mm) between the control (81.65 ± 3.91 mm) and bilateral (75.95 ± 5.68 mm) groups (Table 5; Fig. 5b).

For quantitative dental measurements, the lowest significant difference was for the CW measurement (0.35 mm) between the control (7.77 ± 0.90 mm) and the bilateral (8.12 ± 0.57 mm) groups, and the highest significant difference was for the CL measurement (3.07 mm) between the control (24.98 ± 2.59 mm) and unilateral (21.91 ± 2.15 mm) groups. There was a significant difference in the measurement length and width of canines among the impaction and control groups (*p* < 0.001). The impacted canines (unilateral and bilateral) were significantly shorter and wider than those of the control canines. There were no significant differences between bilateral and unilateral length and width of canine (Table 5; Fig. 5c).

Regarding the surrounding bone density, statistical analysis compared the mean value of the four right sides in control (499.9 ± 143.04 HU) and unilateral (765.69 ± 156.87 HU) or bilateral (751.78 ± 135.67 HU), and the mean value of the four left sides in control (593.63 ± 124.71 HU) and unilateral (842.96 ± 126.31 HU) or bilateral (806.19 ± 132.60 HU). Impacted canine groups showed that the bone density area was increased in the impacted canine groups compared to their normal peers. There was no significant difference between the unilateral and bilateral groups. There was no significant difference between right and left bone density (Table 6; Fig. 5d).

**Table 6:** Comparison of qualitative measurements between control and impaction groups

	Bone density Mean ± SD (Min, Max), HU			p- value*
	Control	Unilateral	Bilateral	
Right all surface	499.9 ± 143.04 (102, 759)^a^	765.69 ± 156.87 (412, 1016)^b^	751.78 ± 135.67 (481, 1020)^b^	0.000^*^
Right				
B	316 ± 211.70 (46, 947)^a^	707.68 ± 272.22 (289,1068)^b^	767.20 ± 252.84 (158, 1351)^b^	0.000^*^
P	566.7 ± 302.19 (76, 989)^a^	774.16 ± 222.59 (290, 1133)^b^	749.98 ± 206.92 (111,1166)^b^	0.000^*^
M	538.16 ± 316.2 (76, 968)^a^	767.53 ± 242.87 (300, 1125)^b^	749.18 ± 206.95 (111, 1032)^b^	0.000^*^
D	578.76 ± 231.42 (111, 881)^a^	813.42 ± 160.31 (528, 1102)^b^	740.76 ± 237.74 (59, 1246)^b^	0.000^*^
Left all surface	593.63 ± 124.71 (357, 863)^a^	842.96 ± 126.31 (569, 1054)^b^	806.19 ± 132.60 (428, 1046)^b^	0.000^*^
Left				
B	458.38 ± 215.79 (90, 1027)^a^	809.77 ± 232.59 (435, 1257)^b^	815.88 ± 244.86 (204, 1359)^b^	0.000^*^
P	595.08 ± 273.46 (97, 952)^a^	812.97 ± 175.31 (275, 1059)^b^	809.58 ± 201.34 (311, 1136)^b^	0.000^*^
M	599.58 ± 258.61 (141, 970)^a^	855.48 ± 159.40 (525, 1229)^b^	771.86 ± 204.22 (274, 1127)^b^	0.000^*^
D	721.50 ± 180.89 (338, 1092)^a^	893.65 ± 188.70 (539, 1175)^b^	827.46 ± 206.59 (357, 1331)^b^	0.001^*^

^a, b^ Letters used for significance differences between studied groups; similar letter indicated insignificant difference while different letters indicated significant differences between studied groups

* indicates p- value

## Discussion

Different diagnostic methods have been used to investigate aspects connected to canine impaction over the years [15, 21], including plaster casts and orthopantomography. Pulp necrosis, ankylosis, and external apical root resorption can all result from delayed tooth eruption. When resorption will begin is impossible to prevent. As a result, all impacted teeth should be thought of as having a high chance of causing harm to the adjacent tooth or external apical root resorption. Therefore, a radiographic examination is usually needed to monitor these risks. Early detection is necessary to avoid potential problems, especially resorption of the root of adjacent teeth [22].

This study aimed to compare and contrast the qualitative and quantitative evaluation of maxillary basal, dentoalveolar, and dental dimensions in patients with and without maxillary impacted canines to better understand the mechanical environment at the non-impacted and impacted sites. The basal dimension explained the base of the maxillary arch which if reduced usually needs either rapid palatal expansion at early age or surgically assisted rapid palatal expansion during adulthood, while dentoalveolar dimension is limited to the teeth-bearing area that requires dentoalveolar expansion by dentoalveolar expansion mechanics.

Our findings reported statistically differences for the quantitative measurements involving the two basal variables (MBW and PMBW) and all measured dentoalveolar variables. Unilateral and bilateral impacted groups showed significantly wider and shorter canines than the control group. The qualitative measurements (the four bone density areas) around unilateral and bilateral impacted groups showed significantly greater density than the control group. There was no significant qualitative or quantitative difference between the unilateral and bilateral impacted canines. The three studied groups had no significant variations in terms of AD.

Numerous studies in the literature have evaluated bone and dental features in individuals with impacted canines and compared to non-impacted group [23–25]. However, fewer studies have included a control group [15, 26]. Additionally, none of these studies have compared the bilateral group to the unilateral group within the same study or the study group to the control group as examined in the current investigation using a retrospective analysis.

CBCT is an effective method for studying impacted canines [23, 27, 28]. The most accurate diagnostic method for locating impacted teeth is CBCT, which has a lower exposure dosage, lesser cost, and greater image accuracy than traditional CT. It also eliminates the image blurring, overlapping nearby structures, and superimpositions common in panoramic radiography.

Bone trabeculation can be assessed by several means ranging from the periapical radiographs up to the multi-spiral computed tomography [29]. The more sensitive measurement is the bone mineral density which can be accurately measured using CT; but it is costly in terms of radiation exposure, so the other alternative is the use of CBCT; the connection between HU and CBCT values is high when measured bone density at the impacted and erupted canine [30]. On the other hand, this imaging technique has its own drawbacks in this regard; the HU values of subjects are not consistent between different CT systems and between different times scanned even using the same CT system. These discrepancies can arise from the non-uniform process of scaling the HU values during reconstruction [31].

The current findings were in agreement with Sukhia et al. [32, 33] who concluded that Asians have a higher rate of maxillary underdevelopment and anterior transverse deficiency than white people, and Chinese patients had more labially impacted canines especially female patients [34–37].

This study reported a significant reduction in maxillary basal width at two levels (MBW and PMBW) in the impacted groups. These results agreed with Franchesca et al. [38] who reported that the width from the central raphe to the first premolar is narrower on the side of maxillary palatally impacted canines than on the other side of non-impacted canines. The results also agreed with Tadinada et al. [23], who reported on the impacted side: the alveolar bone height and buccopalatal width were significantly reduced. The population similarity might be behind this agreement. The AD was found to be non-significantly different between the impaction groups (unilateral and bilateral) and the control group in this study. These findings corroborated those of Fattahi et al. [39] and Cacciatore et al. [40], who reported equivalent AD in impacted canines, whether buccal or palatal, and in matched controls. The differences might be attributed to the different measurement method used; in their studies they used the physical study model versus the three-dimensional imaging method in the current study.

Our findings reported reductions in maxillary dentoalveolar width at two levels (MAW and PMAW) in the impacted groups. These results agreed with other authors [23, 38] who reported that the alveolar bone dimensions were significantly reduced on the impacted side. This could indicate that smaller maxillary width dimensions are associated with a higher risk of impaction due to limited space in the dental arch. The maxillary width deficit can be diagnosed at a young age (between 8 and 10 years), and interceptive treatment can be done at earlier time points to prevent this problem [41]. Girls' maxillary canines usually erupt at the age of 10.5 years, whereas boys' maxillary canines usually erupt at the age of 11.5 years (with the individual variation of 3–4 years) [42]; the proper timing of maxillary arch expansion procedure could be selected to rectify the transverse deficiency and reduce the risk of canine impaction [43].

The current study reported that unilateral and bilateral impacted maxillary canine groups had considerably smaller IMW, IPW, ICW, and AL than the control group. These findings were consistent with other reports, which reported that the impacted maxillary canine group had a shorter and narrower maxillary arch than the control group [40, 44].

The arch perimeter of the impacted group is lower than the non-impacted groups, consistent with Tadinada et al. [23], who reported on unilateral palatally impacted canines. There was a significant decrease in the arch perimeter on the impacted side; this is mostly due to the reduction in the transverse dimensions at different levels.

The current study found that impacted canines have a shorter length and wider mesiodistal width than the control group; this is most likely produced by interproximal attrition following the canine eruption, consistent with Schmidt et al. [45], who reported that the roots of impacted canines and lateral incisors were smaller than those of contralateral teeth utilized as controls on periapical radiographs of individuals with palatally displaced canines treated with an open surgical exposure and levelling technique.

The results of this study showed that the bone density was increased in the impacted canine group with no significant difference between the unilateral and bilateral impacted canines groups. The difference in bone density between the non-impacted and impacted groups supported the hypothesis that the increased bone density leads to the retardation of the normal development of the growing canine and subsequently failure of normal eruption process [46, 47]. As a result, we hypothesized that increased bone density could have a role in the impaction of buccal and palatal maxillary canines on a local level. This study is helpful in understanding how bone density influences the anchoring of the dental piece and is especially valuable during the treatment-planning process. There were no significant variations between the unilateral and bilateral groups for all qualitative and quantitative measurements.

The null hypotheses were rejected as the authors found significant differences in the qualitative and quantitative maxillary basal, dentoalveolar, and dental dimensions between patients with unilateral or bilateral maxillary impacted canines relative to their normal peers.

One of the limitations of this study was the sample size. Larger sample size is recommended with more emphasis on the comparisons between males and females in all studied variables. Also, more classification based on facial pattern (brachy, mesocephalic, or dolichocephalic) is recommended to evaluate any relation in this regard. Another limitation is that the ethnicity was limited to Han Chinese; the results may change if other ethnicities are involved.

## Conclusion

- The main take-home message is that the qualitative and quantitative significant differences between the maxillary impacted canines and control groups indicated that the early correction of skeletal discrepancy by slow or rapid expansion and/or the early maxillary dentoalveolar expansion by dentoalveolar expansion mechanics can minimize the possibility of impacted maxillary canines.
- Maxillary unilateral or bilateral canine impaction is associated with reduced maxillary basal dimensions except for the arch depth.
- The impacted canines have shorter length and wider mesiodistal dimension compared to the control group.
- The quality of the surrounding bone around the impacted canines is high and might be a causative factor for possible impaction.
- Unilateral and bilateral impactions have the same qualitative and quantitative measurements at all levels.

## Data Availability

Not applicable.
